# Real-Life in Cystic Fibrosis Pediatric Patients Treated With Kaftrio: A Descriptive Observational Study

**DOI:** 10.1177/00099228251330121

**Published:** 2025-04-12

**Authors:** Francisco José García Díaz, María Moreno Ortega, Marcos Medina Bethencourt, María Esther Quintana Gallego, Laura Carrasco Hernández, Carmen Delgado Pecellín, Isabel Delgado Pecellín

**Affiliations:** 1 Servicio de Pediatría, Hospital Universitario Virgen del Rocío, Sevilla, España; 2 Unidad de Fibrosis Quística, Hospital Universitario Virgen del Rocío, Sevilla, España; 3 Unidad de Metabolopatías, Hospital Universitario Virgen del Rocío, Sevilla, España

**Keywords:** cystic fibrosis, Kaftrio, FEV1, sweat chloride, transaminase

## Abstract

In 2020, Kaftrio, a combination of Elexacaftor, Tezacaftor, and Ivacaftor, gained approval for treating cystic fibrosis (CF) in patients from the age of 12 years. This study aims to analyze 1 year of treatment with Kaftrio in pediatric patients, comparing their clinical characteristics with pre-treatment data. This is an observational, descriptive, and longitudinal study in patients with CF older than 12 years with at least 1 F508del mutation treated with Kaftrio for 1 year. Forced expiratory volume in 1 second (FEV1) z-score increased by +1.1 (95% confidence interval [CI] = 0.55 to 1.64), forced vital capacity (FVC) by +0.56 (95% CI = 0.10 to 1.04), and maximal mid-expiratory flow (MMEF) 25/75 improved by +1.53 (95% CI = 0.59 to 2.47). In addition, a reduction of 25.50 points (95% CI = −37.95 to −13.06) in sweat chloride levels was observed. Body mass index (BMI)-for-age z-score (WHO 2006/2007) increased +0.39 (95% CI = 0.02 to 0.77). A transient increase in cough and secretions was noted in 61.53% after starting treatment. Kaftrio improves lung function and BMI and also reduces respiratory exacerbations and sweat chloride levels.

## Introduction

Cystic fibrosis (CF) is a monogenic, autosomal recessive disease caused by mutations in the gene encoding the cystic fibrosis transmembrane conductance regulator (CFTR) protein.^1^ This gene is located on the long arm of chromosome 7 at position 31.2 (7q31.2) and encodes a protein of the same name (CFTR) that functions as an ion channel regulating chloride and bicarbonate transport across the luminal surface of epithelial cells in multiple organs.^1,2^ Defective CFTR function causes multisystem disease, resulting in symptoms at several levels: upper and lower respiratory tract, gastrointestinal tract, liver, pancreas, and reproductive system, with lung disease nowadays being the main determinant of associated morbidity and mortality.^3^

There are more than 2000 mutations in the CFTR gene, which are grouped into 7 classes based on the molecular consequences on the CFTR protein: no production (class I), altered protein transport/maturation (class II), activation defects (class III), conduction defects (class IV), reduced production (class V), defects in protein stability at the apical membrane (class VI), and lack of mRNA transcription (class VII).^1,2,4,5^ The most prevalent CF-causing mutation involves the deletion of a phenylalanine at position 508 (F508del) and affects approximately 80% of cases worldwide.^6^ This mutation causes incomplete folding of the protein (class II), but it is also associated with activation defects (class III) and reduced cell surface permanence (class VI).^5^

New modulator drugs act directly on primary CFTR defects.^2^ There are 2 main types: potentiators, which increase the time the CFTR channel is kept open and improve defects in CFTR activation; and correctors, which improve the trafficking of CFTR to the cell membrane when there is a misfolding problem that causes it to degrade prematurely.^7^ Currently, the CFTR modulators approved for clinical use by both the Food and Drug Administration (FDA) and the European Medicines Agency (EMA) are the potentiator ivacaftor (IVA, VX-770) and the correctors lumacaftor (LUMA, VX-809), tezacaftor (TEZA, VX-661), and elexacaftor (ELEXA, VX-445).^2^

The combination of Elexacaftor, Tezacaftor, and Ivacaftor known as Kaftrio in Europe or Trikafta in the United States was approved for use from 12 years old in 2020 and from 6 years of age in 2022.^7^ The triple combination has shown a significant improvement in sweat chloride, lung function, weight and nutritional indexes, a decrease in respiratory exacerbations, and improved quality of life.^8^

It is critical to extend research on the drug Kaftrio to include data from pediatric and adolescent patients, as most studies have focused on the adult population. The objective of this study is to describe the clinical characteristics of a sample of pediatric patients after 1 year of treatment with Kaftrio compared to 1 year prior to the start of treatment.

## Materials and Methods

This is an observational, descriptive, and longitudinal study of pediatric patients with CF treated with Kaftrio during 1 year. Patients being followed up in the CF unit of a tertiary hospital who started treatment with Kaftrio during the first 4 months of 2022 were included in the study. Patients had to be 12 years of age or older at the start of treatment with at least 1 allele with the F508del mutation. Patients (36) who had previously received treatment with a modulatory drug were excluded. After applying the inclusion and exclusion criteria, the final sample size was 13 patients. Follow-up was performed for 1 year, and there were no losses during this period.

For each patient, the following variables were recorded in a database at baseline and after completing 1 year of treatment: age in years, sex, weight in kilograms (kg), height in centimeters (cm), body mass index (BMI) in kg/m^2^ and BMI-for age z-score (OMS 2006/2007), mutations, pancreatic function (sufficient/insufficient), sweat chloride in mmol/L, spirometric values (forced expiratory volume in 1 second [FEV1], forced vital capacity [FVC], maximal mid-expiratory flow [MMEF] 25/75) expressed in liters (L), theoretical percentage (%) and z-score, transaminases in U/L, bilirubin in mg/dL, respiratory colonization, respiratory exacerbations (describing the requirement for oral and intravenous antibiotic treatment and hospital admission), the development of allergic bronchopulmonary aspergillosis, inclusion on the transplant list, and the presence of digestive complications and possible side effects of Kaftrio.

Data were collected in a database, and statistical analysis was performed using IBM SPSS Statistics version 25.0. Categorical variables were expressed as frequency (absolute numbers) and proportions (percentages), and continuous variables were described by median or mean ± standard deviation. A repeated measures study analyzed using an analysis of variance (ANOVA) test was performed to compare the difference between variables before and at 1 year of treatment.

This study was approved by the Provincial Drug Research Ethics Committee of Seville (approval no. 0950-N-23) on June 29, 2023.

## Results

During the study, the above-mentioned variables were analyzed over 12 months of treatment in a total of 13 patients: 8 males (61.50%) and 5 females (38.50%). The average age at the start of treatment was 15.29 years (±1.81 SD).

All patients had the F508del mutation in heterozygosis with one of the following mutations, in order of highest to lowest frequency: R334W (30.80%), G542X (23.10%), N1303K (15.14%), C.695 T>A (7.69%), C.489+1G>T;621+1G>3 (7.69%), S1045Y (7.69%), and C.579+1G>T;711+1G>T (7.69%).

Regarding anthropometric variables, the mean weight at the start of treatment was 49.35 kg (±6.68 SD), increasing to 55.71 kg (±6.33 SD), corresponding to an increase of +6.36 kg (95% confidence interval [CI] = 4.37 to 8.34, *P* < .001). The mean pre-treatment BMI was 18.81 (±1.63 SD), rising to 20.23 (±1.50 SD) after 12 months, an increase of +1.42 (95% CI = 0.67 to 2.16, *P* = .001). The BMI-for-age z-score (WHO 2006/2007) was −0.52 (±0.76 SD) prior to treatment and −0.13 (±0.61 SD) after 12 months of treatment, reflecting an increase of +0.39 (95% CI = 0.02 to 0.77, *P* = .04).

Concerning lung function assessment parameters: the mean pre-treatment FEV1 value was 2.87 L (±0.61 SD) compared to 3.56 L (±0.59 SD) after 1 year of treatment, a difference of +0.69 L (95% CI = 0.46 to 0.91, *P* = .001). The mean increase in FEV1 percentage was +13.76% (95% CI = 6.51 to 21.02, *P* = .001). Regarding the FEV1 z-score, the mean value prior to treatment was −1.04 (±1.12 SD), increasing to +0.06 (±0.85 SD) after 1 year of treatment, representing an increase of +1.1 (95% CI = 0.55 to 1.64, *P* < .001). The mean FVC was 3.69 L (±0.64 SD) vs 4.29 L (±0.62 SD), with an increase of +0.60 L (95% CI = 0.41 to 0.78, *P* = .001) and +8.30% (95% CI = 2.90 to 13.71, *P* = .006). The mean FVC z-score prior to treatment was −0.16 (±0.82 SD), increasing to +0.40 (±0.89 SD) after 1 year of treatment, reflecting an increase of +0.56 (95% CI = 0.10 to 1.04, *P* = .02). For MMEF 25/75, there was an increase at 1 year of treatment of +0.33 L (95% CI = 0.66 to 2.12, *P* = .001), an improvement of +24.53% (95% CI = 12.21 to 36.86, *P* = .001), and a rise in the z-score of +1.53 (95% CI: 0.59 to 2.47, *P* = .004). Table 1 summarizes the values of the spirometric variables analyzed in each patient.

**Table 1.** Spirometric Parameters.

Patient	FEV_1_ L (%) z-score		FVC L (%) z-score		MMEF 25-75 L (%) z-score	
	Pretreatment	After 12 months	Pretreatment	After 12 months	Pretreatment	After 12 months
1	2.06 (61) −3.17	3.18 (86) −1.21	2.92 (76) −2.23	4.13 (96) −0.41	1.20 (32) −3.89	2.82 (68) −1.57
2	2.45 (82) −1.38	3.24 (108) +0.71	3.37 (102) +0.26	3.82 (104) +1.12	1.77 (47) −2.64	3.92 (86) +0.29
3	2.83 (103) +0.14	3.54 (115) +1.27	3.35 (104) +0.21	4.32 (120) +1.75	2.87 (92) −0.43	3.37 (96) −0.17
4	2.98 (87) −0.92	3.31 (95) +0.64	3.74 (96) −0.09	4.24 (107) +0.56	2.75 (68) −1.52	3.14 (77) −1.11
5	3.07 (82) −1.20	3.97 (102) +0.17	4.74 (110) +1.33	5.21 (117) +1.37	1.90 (46) −2.82	3.38 (76) −1.10
6	2.96 (78) −1.68	4.34 (110) +0.80	4.44 (102) +0.40	5.37 (118) +1.42	1.62 (37) −3.34	4.54 (101) +0.03
7	3.50 (90) −0.46	4.52 (108) +0.71	4.39 (96) +0.15	5.00 (102) +0.24	3.77 (76) −0.41	6.94 (105) +2.04
8	3.72 (93) −0.41	3.95 (92) −0.64	4.34 (94) −0.34	4.52 (91) −0.78	3.14 (92) −0.26	4.28 (90) −0.44
9	3.58 (106) +0.51	3.80 (111) +0.79	3.74 (98) −0.14	4.01 (107) +0.14	6.08 (152) +2.31	5.38 (134) +1.43
10	1.81 (61) −3.16	2.52 (83) −1.43	2.98 (90) −0.73	3.71 (110) +0.77	0.90 (25) −4.07	3.68 (71) −0.04
11	2.21 (88) −0.94	2.58 (95) −0.27	2.63 (94) −0.38	3.12 (104) +0.46	2.41 (74) −1.21	4.46 (95) +1.42
12	3.49 (104) −0.08	3.76 (93) −0.56	3.78 (97) −0.70	4.04 (86) −1.22	4.25 (112) +0.33	4.59 (102) +0.13
13	2.75 (89) −0.78	3.67 (105) −0.22	3.63 (101) +0.12	4.39 (106) −0.16	2.13 (62) −1.83	3.42 (89) −0.77

The total number of respiratory exacerbations (Table 2) in the year prior to treatment was 31 (2.38 exacerbations/patient), decreasing to 14 (1.08 exacerbations/patient) after 12 months on Kaftrio.

**Table 2.** Respiratory Exacerbations.

Patient	Number of exacerbations		Cycle of oral antibiotic therapy		Cycle of antibiotherapy intravenous		Admissions	
	Pretreatment	After 12 months	Pretreatment	After 12 months	Pretreatment	After 12 months	Pretreatment	After 12 months
1	3	1	2	0	1	0	1	0
2	6	1	3	0	3	0	0	0
3	1	1	2	1	0	0	0	0
4	2	1	1	1	1	0	0	0
5	0	2	0	1	0	0	0	0
6	5	1	5	0	0	0	0	0
7	1	0	0	0	0	0	0	0
8	4	0	2	0	0	0	0	0
9	2	1	2	0	0	0	0	0
10	2	1	1	1	1	0	1	0
11	3	1	3	0	0	0	0	0
12	1	2	1	2	0	0	0	0
13	1	2	1	2	0	0	0	0
Total	31	14	23	8	6	0	2	0

The number of oral antibiotic therapy cycles decreased from 23 to 8 and the number of intravenous antibiotic therapy cycles from 6 to 0. Two patients required hospital admission in the year prior to treatment compared to no admissions after starting treatment.

In the year prior to the initiation of treatment with Kaftrio, 38.46% of patients presented with chronic colonizations, distributed as follows: 5 cases of methicillin-sensitive *Staphylococcus aureus* (MSSA), 1 case of *Pseudomonas aeruginosa*, and 1 case of *Achromobacter xylosoxidans*. During the year of treatment, 5 cases of chronic colonization by MSSA were observed, of which 3 corresponded to previously colonized patients and 2 were new cases. In addition, chronic colonizations by *P aeruginosa* and *A xylosoxidans* were completely eradicated, as well as in 2 patients previously colonized by MSSA.

The liver function markers analyzed in the study were aspartate aminotransferase (AST), alanine aminotransferase (ALT), and total bilirubin. The ALT values >40 U/L in males and >35 U/L in females, AST values >40 U/L in both sexes, and bilirubin values >1 mg/dL were considered pathological. A total of 38.46% of patients had pathological ALT values at baseline, compared to 7.69% at 1 year of treatment. Pathological AST values were present in 23.07% of patients at baseline compared to 7.69% at 1 year. Concerning bilirubin values, 7.69% of patients had pathological values pre-treatment compared to 15.38% after 1 year of treatment.

In terms of pancreatic function, 46.15% of patients had pancreatic sufficiency (defined by pancreatic elastase levels >200 mcg/g), and after 1 year, the percentage of patients with pancreatic sufficiency was 38.46%. This means that only 1 patient developed pancreatic insufficiency during the year of treatment, representing a new diagnosis, as this patient had pancreatic sufficiency at baseline.

The sweat chloride test was positive in all patients at the start of treatment with a mean sweat chloride value of 88.97 mmol/L. After 1 year of treatment with Kaftrio, 11 patients showed a decrease in chloride levels, 1 patient had no change in chloride levels, and 1 patient showed an increase in chloride levels. Of the 11 patients who showed a decrease, 2 of them reduced their levels to below 30 mmol/L, which is a sweat test negative; in 4 of them, the values decreased to a range between 30 and 59 mmol/L; and in the remaining patients, the values decreased but did not fall below 60 mmol/L. On average, sweat chlorine levels decreased by 25.50 points (95% CI = −37.95 to −13.06, *P* = .001).

In the year previous to starting treatment, there were 2 cases of allergic bronchopulmonary aspergillosis, while there were no new cases after starting Kaftrio. There were no patients on the transplant list either at the start of treatment or after the first year of treatment.

Prior to the beginning of therapy, 4 patients (30.77%) suffered from digestive complications: 1 patient had liver cirrhosis and portal hypertension, 1 patient had an episode of acute pancreatitis, 1 patient developed recurrent pancreatitis, and 1 patient developed acute peritonitis. During the year of treatment, 1 patient (7.69%) developed intestinal obstruction.

Regarding possible side effects, in the first weeks after starting treatment with Kaftrio: 8 patients (61.53%) reported a temporary increase in cough and secretions; 1 patient reported an increased appetite; and another patient had a pruritic rash on the soles of the feet. Finally, 3 patients had self-limited episodes of headache, abdominal pain, and asthenia, respectively. None of these symptoms required discontinuation of treatment.

## Discussion

The approval of new CFTR modulators has brought about a radical change in the treatment of CF patients.^6^ In particular, the available experience with the triple therapy consisting of Elexacaftor, Tezacaftor, and Ivacaftor shows encouraging results in terms of improvement in several aspects of the disease compared to other modulators.^9^

The impact on lung function has been studied by many authors.^10,11^ In our case, we analyzed the effect of Kaftrio on FEV1, FVC, and MMEF 25/75 values. There was an increase of +0.69 L (+13.76%) in FEV1 and an increase of +0.60 L (+8.30%) in FVC, as well as an increase of +1.1 y +0.56 in the z-score, respectively. Nichols et al^12^ report a mean increase in FEV1 of 9.8% at 6 months of treatment, which is slightly higher (+10.8%) in the subgroup of patients who had not received prior treatment with modulatory drugs. Regarding FVC, Salvatore et al^13^ found an increase of 19.27% after 24 weeks of treatment. We have not found a common record of changes in MMEF 25/75 in the studies reviewed; however, in our analysis, we observed a mean increase of +0.33 L (+24.53%). In this regard, we believe that further studies analyzing these parameters would be useful to provide more robust support for the beneficial effect of this drug. However, the variability and lack of consensus regarding the interpretation of MMEF 25/75 values have contributed to its progressively decreasing use in routine clinical practice.^14^

The Kaftrio therapy has also been associated with a reduction in the number of respiratory exacerbations.^10,15,16^ In our study, the annual exacerbation rate decreased by 54.84% during the year of treatment compared to the previous year without treatment. Similar data were found by Middleton et al^17^ with an annual rate of pulmonary exacerbations 63% lower than with placebo. Furthermore, in addition to a reduction in the number of respiratory exacerbations, a reduction in the need for cycles of antibiotic therapy (oral or intravenous) has also been observed, as well as a reduction in the need for hospital admission.^10^ In our sample, a reduction in the annual antibiotherapy rate of 72.41% was observed, similar to those reported by Salvatore et al^13^ with an 80% antibiotherapy reduction rate. Miller et al^16^ also describe a decrease in the number of antibiotherapy days within 15 weeks of starting Kaftrio compared to untreated patients. Moreover, in the study by Salvatore et al,^13^ no admissions were recorded during the course of Kaftrio treatment for 24 weeks, compared to 17 hospitalizations in the same group of patients during the 24 weeks prior to initiation of treatment. In our study, no hospital admissions for respiratory exacerbations were necessary in contrast to 2 admissions during the previous year.

As for nutritional parameters, one of the effects described in different studies during Kaftrio treatment is an increase in weight and BMI.^18^ In our research, we observed an increase of 1.42 points in BMI after 1 year of treatment, similar to those published by Middleton et al^17^ with a mean gain of 1.04 points after 24 weeks of treatment in patients ≥12 years compared to placebo.

On sweat chloride concentration, Zemanick et al^19^ carried out a study on the effect of Kaftrio after 24 weeks of treatment in children aged 6 to 11 years, observing a greater decrease in sweat chloride concentration than that described in other studies of adults and adolescents, with a mean decrease of −60.9 mmol/L,^19^ compared to Nichols et al^12^ who observed a mean reduction of −41.7 mmol/L in patients aged ≥12 years. Moreover, the decrease was even more pronounced in the group of patients with homozygous F508del, with a mean decrease of −70.4 mmol/L.^19^ In our study, the results were not as striking, with a mean decrease of −25.50 mmol/L after 1 year of treatment. However, in 15.38% of patients, values below 40 mmol/L were obtained, resulting in a negative test, and in 30.77%, values between 40 and 60 mmol/L were obtained, in which case, the test was considered doubtful.

Some studies report a treatment-related increase in aminotransferases.^10^ During 24 weeks of treatment, Middleton et al^17^ describe an increase in AST or ALT in 10.90% of patients on Kaftrio compared to 4% of patients on placebo. Nevertheless, the results of our study show that after 1 year of treatment with Kaftrio, there is a decrease in the number of patients with abnormal transaminase values. In particular, a decrease in aminotransferase levels below the pathological threshold was observed in 30.77% of patients treated with Kaftrio. Perhaps, the elevation of liver enzymes observed in the aforementioned studies may be related to other factors of the disease itself and not only to treatment with Kaftrio.

On the contrary, a slight increase in bilirubin values was observed after starting treatment, an effect also found by Keating et al.^20^ However, in our study, only 7.69% of patients had pathological total bilirubin values 1 year after treatment with Kaftrio. Keating et al^20^ describe an incidence of elevated bilirubin levels more than twice the upper limit of normal in 3% of patients in their study.

With regard to possible side effects of Kaftrio treatment, in our study, 61.54% of patients had increased cough and respiratory secretions in the first weeks of treatment, which disappeared after 2 to 3 weeks; a side effect frequently described in the literature.^20,21^ Skin rash has also been documented as a possible self-limiting side effect that occurs more frequently in women.^17^ Although in most cases it is mild, it has occasionally necessitated discontinuation of treatment.^17,22^ In our case, a male patient presented with pruritic plantar exanthema, which was mild and self-limiting and did not require discontinuation of treatment. Other possible side effects of treatment found in other studies include pyrexia, chest pain, anxiety, and depression.^9,20^ In our patients, headache, abdominal pain, and asthenia were described as symptoms after the start of treatment, without being able to establish a causal relationship between these symptoms and the treatment. As observed in our study, no publication describes deaths during clinical trials to date.^10^

One of the most important limitations of the study is the small sample size and, in addition, the exclusion of patients who received treatment with a modulator drug before the start of the study makes the final number of patients even smaller. Other limitations were the lack of stratification of patient severity according to lung function parameters and the absence of a control group.

## Conclusions

Kaftrio improves lung function significantly, measured both in FEV1 and FVC and in MMEF 25/75. In addition, after the start of treatment, the number of respiratory exacerbations is considerably reduced, and therefore, the need for antibiotic therapy and hospital admission. Furthermore, the improvement in the nutritional profile is evident, with a weight gain that brings BMI values closer to the normal range. It is worth noting the decrease in sweat chloride levels observed after starting treatment with Kaftrio, which in some cases led to a negative sweat test. Finally, as far as side effects are concerned, we have not observed the increase in transaminases described in the literature, although there has been an increase in bilirubin values. The increase in cough and secretion production after immediate drug initiation and the rash seem to be related to the drug, without being able to establish causality. Studies with larger numbers of patients are needed to determine possible side effects in a robust way.

## Author Contributions

GD, MO, MB, QG, CH, DP, DP: Contributed to conception and design; Contributed to analysis; Drafted the manuscript; Critically revised the manuscript; Gave final approval; Agrees to be accountable for all aspects of work ensuring integrity and accuracy.
